# Vitamin D Supplementation and Testosterone Levels in Breast Cancer Survivors

**DOI:** 10.3390/ijms262010030

**Published:** 2025-10-15

**Authors:** Anita Minopoli, Piergiacomo Di Gennaro, Giuseppe Porciello, Elvira Palumbo, Sara Vitale, Maria Grimaldi, Rosa Pica, Luca Falzone, Concetta Montagnese, Renato de Falco, Anna Crispo, Denise Giannascoli, Lucia Di Capua, Serena Meola, Monica Pinto, Michelino De Laurentiis, Vincenzo Di Lauro, Francesco Ferraù, Francesca Catalano, Francesco Messina, Massimiliano D’Aiuto, Massimo Rinaldo, Vincenzo Montesarchio, Davide Gatti, Agostino Steffan, Samuele Massarut, Jerry Polesel, Massimo Libra, Ernesta Cavalcanti, Egidio Celentano, Livia S. A. Augustin

**Affiliations:** 1 Laboratory Medicine Unit, Istituto Nazionale Tumori IRCCS Fondazione Pascale—IRCCS di Napoli, 80131 Naples, Italy; a.minopoli@istitutotumori.na.it (A.M.); renato.defalco@istitutotumori.na.it (R.d.F.); l.dicapua@istitutotumori.na.it (L.D.C.); serena.meola@istitutotumori.na.it (S.M.); e.cavalcanti@istitutotumori.na.it (E.C.); 2 Medical Statistics Unit, University of Campania “Luigi Vanvitelli”, 80138 Naples, Italy; piergiacomo.digennaro@unicampania.it; 3 Epidemiology and Biostatistics Unit, Istituto Nazionale Tumori IRCCS Fondazione Pascale—IRCCS di Napoli, 80131 Naples, Italy; g.porciello@istitutotumori.na.it (G.P.); elvira.palumbo@istitutotumori.na.it (E.P.); sara.vitale@istitutotumori.na.it (S.V.); m.grimaldi@istitutotumori.na.it (M.G.); r.pica@istitutotumori.na.it (R.P.); a.crispo@istitutotumori.na.it (A.C.); e.celentano@istitutotumori.na.it (E.C.); 4 Department of Biomedical and Biotechnological Sciences, Oncologic, Clinical and General Pathology Section, University of Catania, 95124 Catania, Italy; luca.falzone@unict.it (L.F.); m.libra@unict.it (M.L.); 5 Institute of Food Science, CNR Italy, 83100 Avellino, Italy; concetta.montagnese@isa.cnr.it; 6 Division of Radiology, Istituto Nazionale Tumori IRCCS Fondazione Pascale—IRCCS di Napoli, 80131 Naples, Italy; denise.giannascoli@istitutotumori.na.it; 7 Rehabilitation Medicine Unit, Istituto Nazionale Tumori IRCCS Fondazione Pascale—IRCCS di Napoli, 80131 Naples, Italy; m.pinto@istitutotumori.na.it; 8 Department of Breast and Thoracic Oncology, Istituto Nazionale Tumori IRCCS Fondazione Pascale—IRCCS di Napoli, 80131 Naples, Italy; m.delaurentiis@istitutotumori.na.it (M.D.L.); vincenzo.dilauro@istitutotumori.na.it (V.D.L.); 9 Clinical and Translational Oncology, Scuola Superiore Meridionale, 80138 Naples, Italy; 10 Ospedale San Vincenzo, 98039 Taormina, Italy; ferrau@oncologiataormina.it; 11 Cannizzaro Hospital, 95021 Catania, Italy; fcatalano1968@tiscali.it; 12 Ospedale Evangelico Betania, 80147 Naples, Italy; messina52@alice.it; 13 Breast Unit, Ospedale di Boscotrecase, 80042 Boscotrecase, Italy; massimiliano.daiuto@gmail.com (M.D.); massimo.rinaldox@gmail.com (M.R.); 14 UOC Oncologia, AORN dei Colli (Monaldi-Cotugno-CTO), 80131 Naples, Italy; vincenzo.montesarchio@ospedalideicolli.it; 15 Rheumatology Unit, University of Verona, 37129 Verona, Italy; davide.gatti@univr.it; 16 Immunopathology and Cancer Biomarkers Unit, Centro di Riferimento Oncologico di Aviano (CRO), IRCCS, 33081 Aviano, Italy; asteffan@cro.it; 17 Chirurgia Oncologica del Seno—Centro di Riferimento Oncologico di Aviano (CRO), IRCCS, 33081 Aviano, Italy; smassarut@cro.it; 18 Unit of Cancer Epidemiology, Centro di Riferimento Oncologico di Aviano (CRO), IRCCS, 33081 Aviano, Italy; polesel@cro.it

**Keywords:** breast cancer survivors, cholecalciferol (vitamin D), 25-hydroxyvitamin D [25(OH)D], testosterone, sex hormone–binding globulin (SHBG), androgens, hormone-suppressive therapy, aromatase inhibitors, lifestyle intervention, body mass index (BMI)

## Abstract

Vitamin D plays a key role in immune modulation, cell proliferation, and hormone regulation. Dysregulated testosterone may contribute to breast cancer progression. We investigated whether long-term vitamin D supplementation affects serum testosterone levels in breast cancer survivors. Complete data at baseline, 12, and 24 months were derived from 253 women with early-stage breast cancer participating in the DEDiCa trial and randomized to receive either a high-dose vitamin D to maintain serum 25(OH)D at 60 ng/mL (group A) or a standard dose to maintain serum levels at 30 ng/mL (group B). Serum 25(OH)D levels significantly increased in both groups (*p* < 0.001). No significant changes in testosterone concentrations were observed between treatment groups over the 24 month treatment period (A: 0.125 to 0.140 ng/mL; B: 0.162 to 0.193 ng/mL; *p* = 0.682). Baseline serum testosterone levels emerged as the most significant predictor of testosterone trajectories, possibly modulated by hormone-suppressive therapy. These results are reassuring that vitamin D supplementation did not adversely affect testosterone levels in this population of breast cancer survivors and may partially concur with a healthy lifestyle to equilibrate testosterone levels.

## 1. Introduction

Breast cancer represents the most common female cancer and the main cause of cancer deaths in women worldwide [1]. The etiology of breast cancer is driven by complex interactions among genetic predisposition, hormonal influences, and environmental factors, including lifestyle, diet, and exposure to carcinogens [2,3,4,5,6]. In this context, vitamin D plays a regulatory role in hormone balance, immune modulation, and cell growth control. The major circulating metabolite of vitamin D, 25-hydroxyvitamin D [25(OH)D], is widely used as a biomarker of vitamin D status [7]. This fat-soluble vitamin plays a crucial role in maintaining calcium homeostasis, bone health, immune regulation, inflammation, and cell differentiation, and has been linked to lower total and breast cancer-specific mortality [8,9,10].

Sex hormones, including estrogen, progesterone, and testosterone, play a crucial and multifaceted role in breast cancer progression, growth, and prognosis [11,12,13]. Testosterone, primarily known as an androgenic hormone associated with male physiology [14], also plays an important role in female endocrine health. In women, it contributes to various physiological functions, including maintenance of muscle mass, bone density, and libido [15,16]. However, dysregulated testosterone levels in women have been associated with hormone-sensitive cancers, including breast cancer [17,18,19]. In postmenopausal women, testosterone can convert to estrogen via aromatase due to reduced ovarian estrogen production, thereby increasing circulating estrogen levels, a key driver of hormone receptor-positive breast cancer [20,21,22].

Prospective cohort studies suggest that in breast cancer survivors, elevated serum testosterone levels have been associated with increased risk of disease recurrence, distant metastasis, and breast cancer-specific mortality [23,24,25,26,27,28]. Thus, the dual role of testosterone as a precursor to estrogen and as a direct modulator of androgen receptors in breast tissue highlights its potential influence on breast cancer risk [29,30].

In this regard, vitamin D may influence testosterone levels in women by promoting the expression of key enzymes involved in testosterone synthesis [31,32,33] and vitamin D supplementation may increase testosterone levels in women [34,35,36,37,38,39,40,41].

Furthermore, circulating vitamin D levels have been found to directly associate with sex hormone-binding globulin (SHBG) concentrations, which determine testosterone bioavailability [42,43]. These mechanisms may raise concerns in breast cancer survivors, as increased testosterone levels may indirectly elevate estrogen concentrations and worsen disease prognosis [19,44,45].

Furthermore, low serum vitamin D levels, commonly observed in breast cancer survivors, hinder a clear understanding of the effect of supplementation, as both vitamin D deficiency and excess could have opposite effects on hormonal balance and cancer outcomes [46,47]. The interplay between sex hormones and breast cancer underscores the importance of understanding modifiable factors that influence their regulation, including oral vitamin D.

The DEDiCa study (Diet, Exercise, and vitamin D in Cancer) is a randomized controlled trial primarily aimed at reducing the risk of breast cancer recurrence through a lifestyle program inclusive of oral vitamin D supplementation [48]. In our current analysis, we focused on assessing whether vitamin D supplementation may have influenced circulating testosterone concentrations in breast cancer survivors participating in the DEDiCa trial. Additionally, we explored whether other baseline or longitudinal factors might independently influence testosterone variations, in order to better contextualize the potential role of vitamin D.

## 2. Results

### 2.1. Baseline Data

Baseline characteristics, including age (mean 52 ± 9 years), body mass index (BMI) (mean 27 ± 5 kg/m^2^), and waist circumference (mean 94 ± 13 cm), were similar between the groups (*p* > 0.05). Most women were postmenopausal (92%), disease-free at study enrollment, and had a balanced distribution of cancer characteristics at surgery, such as stage and molecular subtypes, across treatment groups. At baseline, serum 25(OH)D levels were below 30 ng/mL in most patients in both study groups. All baseline data are shown in Table 1.

### 2.2. Serum Concentrations of 25(OH)D and Testosterone by Treatment Groups and Follow-Up Time

**Figure 1:**
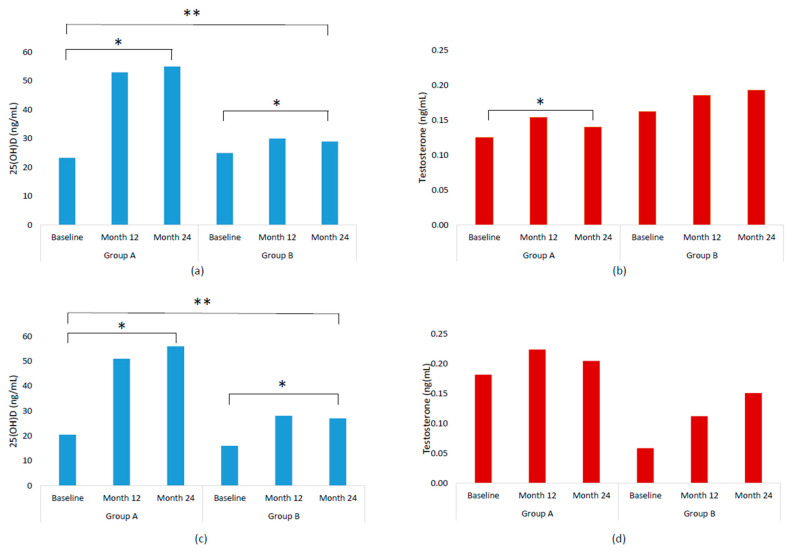
Serum 25(OH)D and testosterone levels (median) by treatment group and time point. (**a**) Serum 25(OH)D levels in total sample; (**b**) Serum testosterone levels in total sample; (**c**) Serum 25(OH)D levels in the subgroup without hormone suppressive therapy; (**d**) Serum testosterone levels in the subgroup without hormone suppressive therapy. * significance (*p* < 0.05) by Wald test for changes at 24 months (mixed model repeated measures—MMRM). ** significance (*p* < 0.05) by Likelihood Ratio Test for time changes between groups (MMRM for group/month interaction adjusted by month).

Table 2 and Figure 1 show serum levels at baseline, 12, and 24 months. Significant increases in serum 25(OH)D levels were observed in both groups over 24 months. In Group A, levels increased significantly from 23 ng/mL to 55 ng/mL (*p* < 0.001) and in Group B from 25 ng/mL to 29 ng/mL (*p* < 0.001) (Table 2). Serum testosterone levels significantly increased over time in group A, rising from 0.125 to 0.140 ng/mL (*p* < 0.05) but not significantly in group B (from 0.162 to 0.193). However, no significant differences were observed between treatments over time (*p* = 0.682). Despite this rise, levels remained within normal ranges and well below clinical thresholds. In the subgroup without hormone-suppressive therapy (*n* = 46), serum 25(OH)D levels increased significantly from 20.5 ng/mL to 56 ng/mL (*p* < 0.001) in group A and from 16 ng/mL to 27 ng/mL in group B (*p* < 0.001) while serum testosterone levels increased from 0.182 to 0.205 ng/mL in group A and from 0.059 to 0.151 ng/mL in group B, although no significant differences were found within each treatment and between treatments (*p* = 0.359).

**Figure 2:**
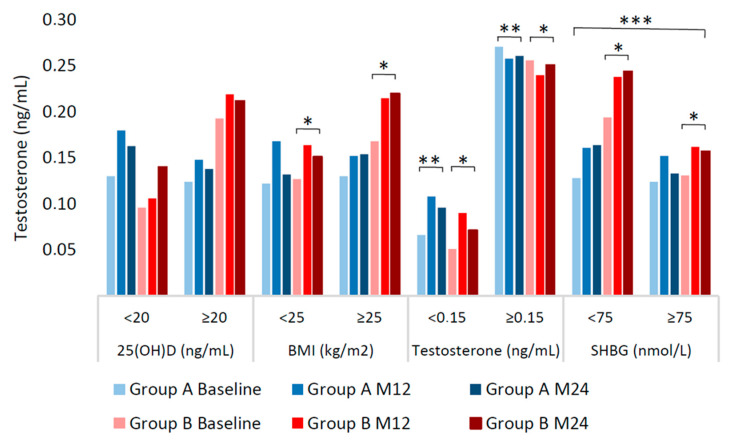
Median testosterone levels by treatment group over time, stratified by selected baseline variables. * Student’s *t*-test with the Satterthwaite method for changes at 24 months by strata within group B (mixed model repeated measures—MMRM). ** Student’s *t*-test with the Satterthwaite method for changes at 24 months by strata within group A (MMRM) *** Wald test by strata between groups on changes at 24 months (MMRM). Abbreviations: M12, month 12; M24, month 24.

Table 3 and Figure 2 show stratified data with the aim of investigating potential effect modifiers. Serum testosterone levels were not different among women stratified by baseline vitamin D levels in either treatment arm (*p* ≥ 0.05) nor between arms (*p* = 0.211). Furthermore, a sub-analysis was conducted on serum testosterone levels in patients with severe baseline vitamin D deficiency (≤10 ng/mL), representing 12% of the overall cohort. In this subgroup, neither changes in testosterone levels over time nor differences between treatments reached statistical significance (Table S1). Among women stratified by baseline testosterone levels, those with lower values (i.e., <0.150 ng/mL) exhibited significant increases in testosterone concentrations over time in both groups (*p* < 0.001) albeit they remained within normal ranges, and no significant differences were observed among strata between groups (*p* = 0.654). In contrast, participants with higher baseline testosterone levels (i.e., ≥0.150 ng/mL) did not experience an increase but a slight, non-significant decline over the study period. Baseline BMI and SHBG emerged as significant effect modifiers of testosterone rises in group B only (*p* = 0.007 and *p* = 0.002, respectively), where patients took lower vitamin D supplementation.

These analyses were designed to clarify whether potential effect modifiers were present in different strata, while the multivariable analysis investigated whether these and other factors exerted an independent effect on testosterone variations.

### 2.3. Multivariable Analysis of Testosterone Variation

Multivariable regression models identified lower baseline testosterone levels as a moderately strong independent predictor of testosterone increments over time (β = −0.37, *p* < 0.001). Baseline SHBG and its variations were inversely but weakly associated with testosterone changes (β = −0.03, *p* = 0.042; β = −0.03, *p* = 0.025, respectively), while there was a significant contribution of BMI increases to testosterone increases (β = 0.69, *p* = 0.039). Other factors, including changes in serum 25(OH)D levels, age, and timing of hormone suppressive therapy, were not significant predictors of testosterone changes (*p* > 0.05) (Table 4). However, hormone suppressive therapy started before baseline suggested a potential effect on testosterone rises (β = −4.3; 95% CI −8.5, −0.07). Therefore a further analysis was performed (i.e., mediation analysis) which showed a significant effect attributable to hormone suppressive therapy mediated by baseline testosterone levels (−0.028, *p* < 0.001). This was approximately 50% of the overall estimated effect (0.48, *p* < 0.001). The average direct effect on testosterone changes attributable to hormone suppressive therapy without taking into account baseline testosterone levels was of borderline significance (−0.031, *p* = 0.055).

## 3. Discussion

In this multicentric randomized trial, we evaluated whether a two-year supplementation with oral vitamin D could significantly affect serum testosterone concentrations in breast cancer survivors. Vitamin D is known to play a relevant role in the synthesis and regulation of female hormones, including sex steroids [49,50,51,52,53]. A cross-sectional study reported a positive correlation between serum vitamin D and testosterone levels in healthy, non-obese women [54]. In contrast, more recently, in a study comparing total and free 25(OH)D levels in healthy women of reproductive age, inverse correlations were found with total testosterone [55]. Overall, our analyses showed no clinically relevant changes nor statistically significant differences in serum testosterone levels between treatment groups following two years of oral cholecalciferol supplementation, despite a significant increase in serum 25(OH)D concentrations. The statistically significant increase in testosterone levels observed in the group treated with higher doses of oral vitamin D (group A), never exceeded the upper limit of normality. Our stratification analysis showed that women with lower baseline testosterone levels had a significant increase in serum testosterone in both treatment groups, suggesting a potential physiological compensatory effect independent of vitamin D supplementation. Conversely, women with higher baseline testosterone levels did not show an increase in testosterone but a tendency for a slight reduction over time, consistent with possible hormonal self-regulatory mechanisms [56]. Multivariate analysis confirmed that the main determinant of longitudinal changes (increases) in serum testosterone concentrations, was represented by its low baseline levels, and a further mediation analysis suggested a 50% contribution of hormone suppressive therapy with low baseline serum testosterone levels. Aromatase inhibitors used as hormone suppressive therapy in women with estrogen-sensitive breast cancer are known to reduce estrogen synthesis from androgens and therefore may increase testosterone levels [57]. In the small subgroup of patients not taking estrogen-suppressive therapy, no relevant testosterone variations were observed, strengthening the null hypothesis of an absent effect of vitamin D on testosterone in the clinical setting of women treated for breast cancer. Variables such as age and vitamin D insufficiency before study treatment were not independent predictors of significant testosterone changes, suggesting that the relationship between vitamin D and sex hormones might be less direct and more complex than previously hypothesized [34,35,36,38,39,40,41,49,55,58,59]. Similarly, also in the subgroup of participants with severe baseline vitamin D deficiency (<10 ng/mL), representing only 12% of our study patients, no significant changes in serum testosterone were observed over time or between treatment groups despite their large increases in serum 25(OH)D. However, this limited number of subjects may have hindered the detection of any meaningful effects of vitamin D supplementation in this specific subgroup. Low levels of 25(OH)D in the insufficiency ranges have been observed in women with polycystic ovary syndrome, clinically characterized also by high testosterone levels, insulin resistance, and excess adiposity [59,60,61,62]. Supplementing these patients with vitamin D reduced total testosterone levels, likely due to improvements in insulin resistance and the overall hormonal environment [60]. An insulin-sensitizing effect of vitamin D cannot be excluded [63]. In our study, we did not observe a significant contribution of insulin resistance (i.e., HOMA-IR) to testosterone rises over time, but BMI increments and SHBG reductions, which are consistent with a condition of insulin resistance, were associated with testosterone rises. Consistently, our multivariable analysis indicated that baseline endocrine and metabolic characteristics—i.e., initial low testosterone and SHBG concentrations, and increasing BMI—were the main independent determinants of testosterone changes, whereas vitamin D supplementation did not emerge as a direct predictor. This supports the interpretation that vitamin D acted more as a contextual factor within the broader lifestyle intervention rather than as an isolated driver of androgen variation.

Our findings may partly reflect the impact of other components of the DEDiCa intervention known to improve glucose metabolism and insulin resistance, specifically the low glycemic index foods and regular brisk walking. Previous studies in women have indeed shown that low glycemic index diets improve insulin sensitivity and are associated with reductions in total serum testosterone concentrations [64,65]. Moderate-intensity physical activity, such as brisk walking, has been associated with improved insulin metabolism and lower androgen levels, potentially through increased SHBG and reduced androgen production [66,67]. While the lifestyle interventions of the DEDiCa trial may have contributed to the observed testosterone modulation, the direct effect of vitamin D on testosterone levels remains unlikely. The broader DEDiCa intervention could have contributed to a physiological adjustment of testosterone levels, which remained within the normal ranges. The possible advantage of normalizing serum testosterone includes preservation of muscle mass and cognitive function [68,69].

A major strength of our study was the uniquely individualized and closely monitored vitamin D supplementation protocol of the randomized trial, which enabled a precise achievement and maintenance of target serum 25(OH)D levels in both intervention arms. This level of monitoring—rarely implemented in vitamin D supplementation studies—minimized the potential for variability due to non-adherence or inconsistent dosing and allowed a reliable assessment of the relationship between vitamin D supplementation and circulating testosterone levels. Additionally, all biochemical analyses were centralized and performed using standardized methods, reducing measurement bias and ensuring high analytical reproducibility. The inclusion of a diverse cohort of breast cancer survivors across multiple Italian centers further strengthens the generalizability of our findings among breast cancer patients. Furthermore, the availability of a biological biobank offers invaluable opportunities for future research, enabling integration of additional serological assessments to further elucidate the complex interplay between vitamin D status, sex hormone dynamics, and clinical outcomes in breast cancer survivors. However, several limitations may be considered. Although the 24-month follow-up may appear long, this timeframe ensured coverage of seasonal vitamin D fluctuations and endocrine changes related to ongoing adjuvant therapy. This is a secondary analysis within a randomized controlled trial of lifestyle treatment, including diet and exercise counseling, together with vitamin D supplementation. Furthermore, our analyses focused on total testosterone concentrations, while measurements of free or bioavailable testosterone and direct androgenic activity were not performed, potentially overlooking subtler endocrine effects mediated through these hormonal fractions. Additionally, although the supplementation and follow-up period were relatively long (24 months), longer-term effects cannot be excluded, e.g., when the endocrine-inhibiting therapy stops after 5–10 years. Despite rigorous monitoring, the observational period may not capture all possible late endocrine or prognostic effects. The strengths and limitations of our study may be considered when interpreting our results and in the design of subsequent studies.

## 4. Materials and Methods

### 4.1. Study Design and Participants

**Figure 3:**
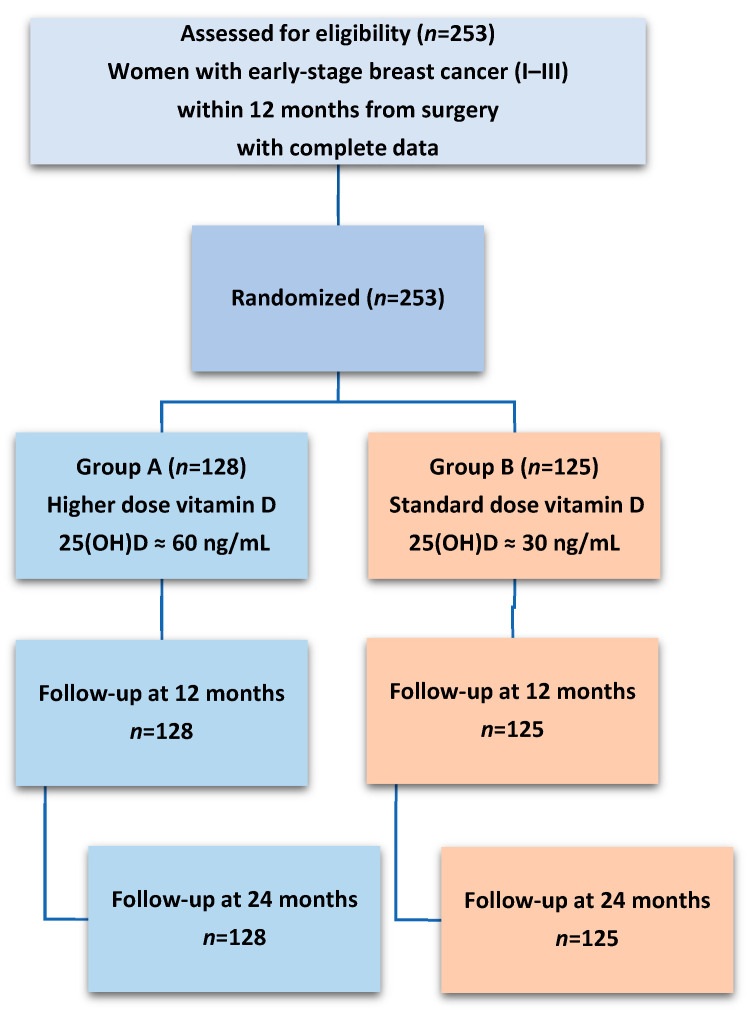
Flowchart of patients in the DEDiCa trial for a subset of participants with complete data used in the current analyses (*n* = 253).

This study was conducted as part of the DEDiCa Trial, a multicenter randomized controlled trial involving hospitals across Italy [48]. The protocol was approved by the Italian Ministry of Health, Italian Medicine Agency—AIFA (EudraCT Number 2015-005147-14) and by the Ethics Committee of each recruiting center (ClinicalTrials.gov identifier NCT02786875). DEDiCa trial investigated the effect of a low glycemic index Mediterranean diet, physical activity, and vitamin D supplementation on breast cancer recurrence. Eligible participants were women aged 30–74 years who had histologically confirmed primary breast cancer (stages I–III according to the TNM staging system), had undergone breast cancer surgery within the previous 12 months, had no evidence of metastatic disease, and had no contraindications for vitamin D supplementation or any components of the lifestyle treatment. Participants were disease-free at study entry, and the cancer stage reported in Table 1 refers to the pathological stage at surgery, which occurred before randomization and was balanced between the two intervention groups. All participants provided written informed consent, and the study protocol was approved by the institutional review boards of each participating site. Participants were randomized into one of two treatment groups. In the high-intensity arm (Group A), participants received structured counseling to follow a low-glycemic-index Mediterranean diet, engage in regular physical activity, and take personalized doses of oral vitamin D (cholecalciferol) to achieve and maintain serum 25(OH)D levels of 60 ng/mL. The low-intensity arm (Group B, positive control) received general advice to adopt a Mediterranean diet and avoid sedentary behavior, along with a standard dose of oral vitamin D to maintain serum 25(OH)D levels at sufficiency (30 ng/mL). The interventions lasted for 33 months. In this analysis, we used complete data from 253 patients (group A: *n* = 128; group B: *n* = 125) at three time points: baseline, 12 months, and 24 months (Figure 3).

The 12- and 24-month follow-up visits were pre-specified in the trial protocol [48] and were chosen for this analysis for several reasons: (1) to encompass at least one full seasonal cycle of serum 25(OH)D, which typically peaks in summer and reaches its nadir in winter [70,71], (2) to reduce the potential influence of an anomalous single year by extending the observation to 24 months [57], and (3) to capture medium-term endocrine changes expected during adjuvant hormone-suppressive therapy and lifestyle interventions. Most patients (*n* = 207) had luminal-type breast cancer and were taking hormone suppressive therapy (i.e., selective estrogen receptor modulators or aromatase inhibitors). Only 46 women were not taking hormone suppressive therapy (group A: *n* = 20; group B: *n* = 26).

### 4.2. Anthropometric and Lifestyle Assessments

Weight and height were measured by trained staff using standardized procedures. Height was measured to the nearest centimeter using a Seca stadiometer, while weight was recorded to the nearest 0.5 kg using the Seca 761 (Seca, Hamburg, Germany). Body mass index (BMI) was calculated as weight (kg)/height (m^2^). Waist circumference was measured to the nearest 1 cm, using a validated non-elastic tape. We gathered data on weekly physical activity using an electronic pedometer (Omron Walking Style IV, OMRON Corporation, Tokyo, Japan), worn by patients for one week before each study visit, and open questions about the type of physical activity.

### 4.3. Biochemical Analyses

Blood samples were collected at baseline, 12 months, and 24 months using a vacuum-based collection system (Vacutainer^®^, BD, Shanghai, China) into serum-separating tubes. After clotting for 30 min at room temperature, samples were centrifuged at 3500 rpm for 15 min. Serum was separated and analyzed immediately after collection on the same day to minimize pre-analytical variability. All biochemical analyses were centralized at the National Cancer Institute—IRCCS “G. Pascale” (Naples). Serum 25(OH)D was quantified by chemiluminescent immunoassay (CLIA, DiaSorin Liaison XL, DiaSorin S.p.A., Vercelli, Italy). Calibration was performed with manufacturer-provided multi-level standards, and internal quality controls at two levels (high and low) were included in each analytical run. The intra- and inter-assay coefficients of variation (CVs) were <8% and <10%, respectively. The assay limit of quantification (LoQ) was 4.0 ng/mL. Institutional reference ranges for cancer patients for 25(OH)D are 30–100 ng/mL, ≤10 ng/mL severe deficiency, >10 <30 ng/mL insufficiency, ≥30 ng/mL sufficiency, and ≥150 ng/mL toxicity. Serum testosterone and sex hormone-binding globulin (SHBG) were measured by electrochemiluminescence immunoassay (ECLIA, Cobas e601/e801, Roche Diagnostics, Mannheim, Germany). The testosterone assay had a LoQ of 0.025 ng/mL, with intra- and inter-assay CVs < 7% and <9%. Age-specific reference ranges were 0.084–0.481 ng/mL for women <50 years and 0.029–0.408 ng/mL for women ≥50 years. The SHBG assay had a LoQ of 0.350 nmol/L, with intra- and inter-assay CVs < 6% and <8%, and reference ranges of 32.4–128.0 nmol/L (<50 years) and 27.1–128.0 nmol/L (≥50 years). For analytical purposes, serum 25(OH)D concentrations were further categorized as ≤10, 10–20, 20–30, or >30 ng/mL.

### 4.4. Statistical Analyses

The general characteristics of the participants were summarized using means and standard deviations for continuous variables and counts with percentages for categorical variables. Menopausal status was classified as either premenopausal or postmenopausal, while smoking status was categorized as current, past, or never. Surgical procedures were classified as either quadrantectomy or mastectomy. Hormonal therapy was reported as the number and percentage of patients undergoing treatment at baseline. Baseline characteristics were analyzed for the overall sample and by randomization group. The analyses by group were implemented using the Wilcoxon rank test for continuous variables and Fisher’s exact test for categorical variables when at least one expected cell count was <5; otherwise, the Pearson’s chi-squared test was used. Univariate analyses were performed for the total sample and for the subgroup of women not receiving hormone-suppressive therapy. Medians, first, and third quantiles were reported separately. Mixed models for repeated measures (MMRM) were used to evaluate changes within groups at month 24 and differences in changes between intervention groups using a Wald test and a Likelihood Ratio Test, respectively.

Subgroup analyses, using MMRM as described above, were conducted on testosterone stratified by baseline serum concentrations of 25(OH)D, testosterone, SHBG, and by BMI categories. A multivariable MMRM was conducted to assess the independent contribution of potential confounders to a 1/100th increase in testosterone levels. Student’s t-test with the Satterthwaite method was performed to test the significance of the coefficients. A mediation analysis was conducted at month 24 to assess the relationship between testosterone levels at baseline and their changes, and hormone suppressive therapy using a bootstrap estimate.

Test results were considered statistically significant with a level of alpha < 0.05. All statistical analyses were performed using R software version 4.4.2.

## 5. Conclusions

In this randomized trial of 253 breast cancer survivors enrolled in the DEDiCa trial, we evaluated the impact of long-term vitamin D supplementation on circulating testosterone levels. Despite achieving and maintaining markedly different serum 25(OH)D targets between intervention arms, no clinically relevant increases in testosterone concentrations were observed over 24 months. Instead, low baseline testosterone and SHBG concentrations, and increasing BMI emerged as the most important determinants of testosterone trajectories, with a potential modulatory role of hormone-suppressive therapy. These findings provide reassurance that higher oral doses of vitamin D, when administered in the context of a healthy lifestyle program, do not adversely influence androgen balance in breast cancer survivors. On the contrary, vitamin D supplementation may contribute indirectly to the maintenance of testosterone levels within physiological ranges, thereby supporting metabolic and endocrine homeostasis.

Future studies should plan to extend follow-up beyond 24 months, include direct measurements of free and bioavailable testosterone, and explore whether vitamin D interacts with endocrine therapies or metabolic profiles in shaping long-term outcomes in breast cancer survivorship.

## Figures and Tables

**Table 1:** Baseline characteristics (mean ± SD or frequencies as % of total subjects) overall (*n* = 254) and by randomization group (group A: *n* = 128; group B: *n* = 125).

	Overall	Group A	Group B	*p*
Age (years), mean ± SD	52 ± 9	52 ± 9	52 ± 9	0.703 ^a^
Waist circ. (cm), mean ± SD	94 ± 13	92 ± 13	95 ± 13	0.155 ^a^
BMI (kg/m^2^), mean ± SD BMI (kg/m^2^)	27 ± 5	27 ± 5	27 ± 5	0.390 ^a^ 0.866 ^b^
<25	112 (44.3%)	56 (43.7%)	56 (44.8%)	
≥25	141 (55.7%)	72 (56.3%)	69 (55.2%)	
25(OH)D ng/mL				0.516 ^b^
≤10	31 (12.3%)	14 (10.9%)	17 (13.6%)	
>10 to 20	63 (24.9%)	32 (25.0%)	31 (24.8%)	
>20 to 30	77 (30.4%)	44 (34.4%)	33 (26.4%)	
>30	82 (32.4%)	38 (29.7%)	44 (35.2%)	
Menopausal status				0.864 ^c^
Post-menopause	230 (90.9%)	116 (90.6%)	114 (91.2%)	
Pre-menopause	21 (8.3%)	11 (8.6%)	10 (8.0%)	
(Missing)	2 (0.8%)	1 (0.8%)	1 (0.8%)	
Smoking status				0.852 ^b^
Never	133 (52.6%)	66 (51.6%)	67 (53.6%)	
Current	43 (17.0%)	21 (16.4%)	22 (17.6%)	
Past	77 (30.4%)	41 (32.0%)	36 (28.8%)	
Type of surgery				0.341 ^c^
Mastectomy	57 (22.5%)	32 (25.0%)	25 (20.0%)	
Quadrantectomy	194 (76.7%)	95 (74.2%)	99 (79.2%)	
(Missing)	2 (0.8%)	1 (0.8%)	1 (0.8%)	
Cancer stage at surgery				0.445 ^c^
I	81 (32.0%)	47 (36.7%)	34 (27.2%)	
IIA	115 (45.4%)	53 (41.4%)	62 (49.6%)	
IIB	29 (11.5%)	13 (10.2%)	16 (12.8%)	
IIIA	22 (8.7%)	11 (8.6%)	11 (8.8%)	
IIIC	6 (2.4%)	4 (3.1%)	2 (1.6%)	
Molecular subtypes				0.156 ^c^
HER2+	11 (4.3%)	3 (2.3%)	8 (6.4%)	
Luminal A	83 (32.8%)	38 (29.7%)	45 (36%)	
Luminal B	127 (50.2%)	72 (56.3%)	55 (44%)	
Triple Negative	32 (12.7%)	15 (11.7%)	17 (13.6%)	
Hormonal therapy use	207 (82%)	108 (84%)	99 (79%)	0.286 ^b^

^a^ Wilcoxon rank sum test; ^b^ Pearson’s Chi-squared test; ^c^ Fisher’s exact test.

**Table 2:** Serum 25(OH)D and testosterone levels (median, Q1–Q3) by treatment group and time point in the total sample and in subgroups without hormone suppressive therapy.

	Group A			Group B			
Variables	Baseline	Month 12	Month 24	Baseline	Month 12	Month 24	*p* ^a^
	*n* = 128	*n* = 128	*n* = 128	*n* = 125	*n* = 125	*n* = 125	
25(OH)D (ng/mL)	23.3 (16.8–32.5)	53.0 (43.8–59.3)	55.0 (46.8–62.0) *	25.0 (15.0–33.0)	30.0 (25.0–34.0)	29.0 (26.0–35.0) *	<0.001
Testosterone (ng/mL)	0.125 (0.05–0.24)	0.154 (0.07–0.26)	0.140 (0.07–0.31) *	0.162 (0.06–0.26)	0.185 (0.08–0.28)	0.193 (0.08–0.28)	0.682
**Patients without hormone suppressive therapy**							
	*n* = 20	*n* = 20	*n* = 20	*n* = 26	*n* = 26	*n* = 26	
25(OH)D (ng/mL)	20.5 (13.3–26.0)	51 (42.5–57.3)	56 (45–63.3) *	16 (12–24)	28 (24–32)	27 (25–30.5) *	<0.001
Testosterone (ng/mL)	0.182 (0.06–0.24)	0.224 (0.08–0.26)	0.205 (0.11–0.28)	0.059 (0.03–0.17)	0.112 (0.04–0.23)	0.151 (0.04–0.26)	0.359

^a^ Likelihood Ratio Test for time changes between groups (mixed model repeated measures—MMRM for group/month interaction adjusted by month), significance *p* < 0.05. * Wald test for changes at 24 months (MMRM), significance *p* < 0.05.

**Table 3:** Median (Q1–Q3) testosterone levels by treatment group over time, stratified by selected baseline variables (*n* = 253).

	Group A					Group B					
		Baseline	M12	M24			Baseline	M12	M24		
	*n* (%)	*n* = 128	*n* = 128	*n* = 128	*p* ^a^	*n* (%)	*n* = 125	*n* = 125	*n* = 125	*p* ^a^	*p* ^b^
25(OH)D (ng/mL)					0.900					0.099	0.211
<20	42 (32.8)	0.126 (0.03–0.22)	0.180 (0.08–0.25)	0.163 (0.07–0.31)		46 (36.8)	0.096 (0.03–0.19)	0.106 (0.03–0.23)	0.140 (0.06–0.24)		
≥20	86 (67.2)	0.124 (0.07–0.25)	0.148 (0.06–0.3)	0.138 (0.06–0.28)		79 (63.2)	0.193 (0.1–0.28)	0.219 (0.14–0.3)	0.212 (0.1–0.29)		
BMI (kg/m^2^)					0.987					0.007	0.056
<25	56 (43.8)	0.122 (0.06–0.27)	0.168 (0.05–0.31)	0.132 (0.06–0.26)		56 (44.8)	0.127 (0.04–0.25)	0.164 (0.06–0.24)	0.151 (0.06–0.24)		
≥25	72 (56.2)	0.130 (0.05–0.23)	0.152 (0.08–0.25)	0.154 (0.08–0.31)		69 (55.2)	0.168 (0.09–0.26)	0.215 (0.11–0.3)	0.220 (0.13–0.34)		
Testosterone (ng/mL)					<0.001					<0.001	0.902
<0.150	74 (57.8)	0.066 (0.03–0.12)	0.108 (0.04–0.17)	0.096 (0.04–0.17)		57 (45.6)	0.051 (0.03–0.1)	0.090 (0.03–0.18)	0.071 (0.04–0.19)		
≥0.150	54 (42.2)	0.271 (0.22–0.32)	0.258 (0.17–0.37)	0.261 (0.12–0.38)		68 (54.4)	0.256 (0.19–0.36)	0.240 (0.18–0.32)	0.251 (0.19–0.34)		
SHBG (nmol/L)					0.615					0.002	0.006
<75	58 (45.3)	0.128 (0.07–0.27)	0.161 (0.09–0.25)	0.164 (0.08–0.31)		68 (54.4)	0.194 (0.09–0.33)	0.238 (0.11–0.32)	0.244 (0.11–0.35)		
≥75	70 (54.7)	0.124 (0.05–0.22)	0.152 (0.06–0.27)	0.133 (0.07–0.26)		57 (45.6)	0.131 (0.04–0.19)	0.162 (0.07–0.22)	0.157 (0.06–0.22)		

Abbreviations: M12, month 12; M24, month 24. ^a^ Student’s *t*-test with the Satterthwaite method for differences between strata within treatment group on changes at 24 months (mixed model repeated measures—MMRM), significance *p* < 0.05. ^b^ Wald test for differences by strata between groups on changes at 24 months (MMRM), significance *p* < 0.05.

**Table 4:** Independent contribution of selected variables to 1/100th unit increase in serum testosterone levels at 24 months in all subjects (*n* = 253) and in women without hormone suppressive therapy (*n* = 46).

	All Subjects (*n* = 253)		
	Beta	95% CI	*p*
Baseline levels			
25(OH)D	0.02	−0.11, 0.14	0.776
Testosterone	−0.37	−0.46, −0.28	<0.001
SHBG	−0.03	−0.07, 0.00	0.042
Baseline changes			
25(OH)D	0.01	−0.06, 0.09	0.704
SHBG	−0.03	−0.06, 0.00	0.025
BMI	0.69	0.03, 1.3	0.039
HOMA-IR			0.145
≤2.5	Ref		
>2.5	−1.7	−3.9, 0.58	
Follow-up			0.634
Month 12	Ref		
Month 24	0.30	−0.95, 1.6	
Age	−0.04	−0.19, 0.11	0.626
Hormone suppressive therapy **			0.121
After baseline (*n* = 35)	Ref		
Before baseline (*n* = 170)	−4.3	−8.5, −0.07	
Never (*n* = 46)	−2.5	−7.3, 2.3	

Derived from Student’s *t*-test with the Satterthwaite method. ** Missing information from 2 subjects.

## Data Availability

The original data presented in this study will be available upon request to the corresponding author and for research purposes only (https://zenodo.org/records/16411561, accessed on 1 September 2025).

## Supplementary Materials

The following supporting information can be downloaded at: https://www.mdpi.com/1422-0067/26/20/10030/s1.

## Abbreviations

The following abbreviations were used in this manuscript:

BMI	Body Mass Index
MMRM	Mixed Model Repeated Measures
SD	Standard Deviation
SHBG	Sex Hormone-Binding Globulin
