# Supplementary material for: Vitamin D Supplementation and Testosterone Levels in Breast Cancer Survivors
Source: Int J Mol Sci. 2025 Oct 15;26(20):10030. doi: 10.3390/ijms262010030 (PMC12564732; doi:10.3390/ijms262010030)
Supplement: Supplementary file 1 [file ijms-26-10030-s001.zip › ijms-3897706-supplementary.pdf]

Table S1. Serum 25(OH)D and testosterone levels (median, Q1–Q3) by treatment group and time point in patients with initial vitamin D deficiency [25(OH)D ≤ 10 ng/mL].

**Total sample**	**Group A**			**Group B**			
**Variables**	**Baseline**	**M12**	**M24**	**Baseline**	**M12**	**M24**	***p***[a]
	n = 14	*n* = 14	*n* = 14	*n* = 17	*n* = 17	*n* = 17	
25(OH)D (ng/mL)	6.7±2.2	47.7±12.0	54±12[b]	8.4±1.4	25.2±7.6	26.5±6.3[b]	<0.001
Testosterone (ng/mL)	0.106 ±0.109	0.120 ±0.123	0.152 ±0.133	0.170 ±0.216	0.170 ±0.157	0.203 ±0.152	0.976

Abbreviations: M12, month 12; M24, month 24.
[a]*p* for Likelihood Ratio Test on time changes between treatment groups (MMRM on group/month interaction), significance $p<0.05$.
[b]*p* for significant likelihood ratio (LR) test on time changes (mixed model repeated measures – MMRM), significance $p<0.05$.
